# New definitions of human lymphoid and follicular cell entities in lymphatic tissue by machine learning

**DOI:** 10.1038/s41598-022-18097-9

**Published:** 2022-11-08

**Authors:** Patrick Wagner, Nils Strodthoff, Patrick Wurzel, Arturo Marban, Sonja Scharf, Hendrik Schäfer, Philipp Seegerer, Andreas Loth, Sylvia Hartmann, Frederick Klauschen, Klaus-Robert Müller, Wojciech Samek, Martin-Leo Hansmann

**Affiliations:** 1 Fraunhofer Heinrich Hertz Institute, Berlin, Germany; 2 TU Berlin, Berlin, Germany; 3 Oldenburg University, Oldenburg, Germany; 4 Frankfurt Institute for Advanced Studies, Frankfurt, Germany; 5 Korea University, Seoul, South Korea; 6 Max-Planck-Institut für Informatik, Saarbrücken, Germany; 7 Goethe University Frankfurt, Molecular Bioinformatics, Frankfurt, Germany; 8 Ludwig-Maximilians-Universität, Munich, Germany; 9 BIFOLD–Berlin Institute for the Foundations of Learning and Data, Berlin, Germany; 10 Charité-Universitätsmedizin, Berlin, Germany; 11 German Cancer Consortium (DKTK), Munich Partner Site, and German Cancer Research Center (DKFZ), Heidelberg, Germany; 12 Senckenberg Institute of Pathology, Goethe University, Frankfurt, Germany; 13 Institute for Pharmacology and Toxicology, Goethe University, Frankfurt, Germany; 14 Department of Otorhinolaryngology and Head and Neck Surgery, Goethe University, Frankfurt, Germany

**Keywords:** Bioinformatics, Time-lapse imaging, Fluorescence imaging, Cellular imaging, Confocal microscopy, Functional clustering, Image processing, Machine learning

## Abstract

Histological sections of the lymphatic system are usually the basis of static (2D) morphological investigations. Here, we performed a dynamic (4D) analysis of human reactive lymphoid tissue using confocal fluorescent laser microscopy in combination with machine learning. Based on tracks for T-cells (CD3), B-cells (CD20), follicular T-helper cells (PD1) and optical flow of follicular dendritic cells (CD35), we put forward the first quantitative analysis of movement-related and morphological parameters within human lymphoid tissue. We identified correlations of follicular dendritic cell movement and the behavior of lymphocytes in the microenvironment. In addition, we investigated the value of movement and/or morphological parameters for a precise definition of cell types (CD clusters). CD-clusters could be determined based on movement and/or morphology. Differentiating between CD3- and CD20 positive cells is most challenging and long term-movement characteristics are indispensable. We propose morphological and movement-related prototypes of cell entities applying machine learning models. Finally, we define beyond CD clusters new subgroups within lymphocyte entities based on long term movement characteristics. In conclusion, we showed that the combination of 4D imaging and machine learning is able to define characteristics of lymphocytes not visible in 2D histology.

## Introduction

The introduction of intravital microscopy with multi-photon technology was an important step for the investigation of motility of lymphoid cells especially in animal models^1^. Many investigations deal with typical B-cell and T-cell reactions like germinal center formation and the impact of cytokines on cell motility^2–5^. Especially follicular T-helper-cells were in the focus of many experiments often in mice models^6^. Genetically modified fluorescent B-cells and T-cells including their specialized subsets could be traced in vivo, mainly in mice models^7–13^. Parameters such as velocities of T-cells and B-cells, continuity of movement, turning angles and others were investigated^13–18^. However, cell movement measurements of immunocompetent cells in human tissues have so far been unavailable due to a lack of appropriate technology^19^. Here, Dijkgraaf et al.^20^ and Donnadieu et al.^21,22^ showed first cellular movements in living human tissue sections. Donnadieu et al. proposed new methods to visualize and track lymphocytes^21^, especially PD1 cells in human germinal centers^22^. In addition, Dijkgraaf et al.^20^ analyzed the motion of CD8 cells in human skin. The motion of CD8 cells in human tissue was compared with the motion of CD8 cells in mice models to rule out technical artifacts caused by e.g. antibodies for immunostaining.

Light microscopic criteria such as nuclear and nucleolar shapes, chromatin distribution, mitotic activity and invasiveness, are established findings for differentiation between different cell types^23,24^. In addition, immunohistochemical and molecular investigations are helpful to define cells and tissues^23,24^. Deep-learning approaches based on convolutional neural networks have been shown to be able to capture rich representations from histopathological data that may remain hidden to the human eye^25–30^. The field of explainable AI (XAI) tries to develop methods to make the decisions of such models understandable (e.g.^31–33^). For applications of XAI in histopathology, see^34,35^. Going beyond the analysis of two-dimensional static images, the analysis of dynamic movies of cell configurations requires computational support^36–40^. There are different approaches, mainly cell tracking, to learn meaningful representations^41^. Alternatively, one can work with the raw movie data either in the form of individual frames or in the form of spatio-temporal patches. The latter approached has not been explored in great depth in the field of histopathology, see^42^. From the methodological point of view, the procedure is closely related to video classification tasks such as human action recognition from natural videos, see^43^. A question that is relevant in action recognition and also central for this work is if the movie provides more information than a collection of slides.

This investigation deals with the incorporation of machine learning, to define and dissect moving immune cells in human reactive lymphoid tissue, to understand human T-cell, B-cell and FDC dynamics and to extend immunohistochemical cell typing by cell morphology and movement. The impact of these new parameters will be tested and compared to conventional approaches.

## Results

In the following, we analyze the movement characteristics from four different angles each discussed in separate sections below: (1) We start with a descriptive analysis of various movement-related and morphological parameters. (2) We discuss in how far this information is sufficient to differentiate between different phenotypes (CD-clusters) in a supervised learning setting. (3) We use the results from the previous steps in conjuction with interpretability methods to identify prototypical properties of different phenotypes, and, finally, (4) we reveal how one can go beyond CD-clusters by differentiating subpopulations based on movement characteristics.

### The motility of lymphocytes in human lymphoid tissue

**Figure 1:**
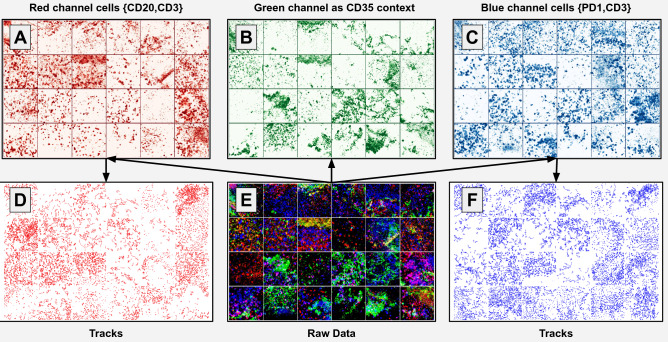
Unique 4D Dataset of lymphocytes inside fresh human lymphoid tissue sections. (**E**) represents the raw data (totaling 24 video sequences where only the first frame is shown). Each sample (i.e.  a single video sequence) consists of three channels (**A**–**C**). Channel B shows CD35 positive follicular dendritic cells, which were used for context analysis. Channel A and C describe a single stain among T-cells (CD3), B-cells (CD20), and follicular T-helper cells (PD1). Moreover, from these channels, cell tracking data is extracted. Specifically, the cell tracks shown in (**D**) and (**F**) are computed from T- and B-cells, respectively. More details are described in “Material and methods”.

For the definition of movement and morphology characteristics^44^, we tracked and evaluated lymphocytes of a unique data-set of B-cells (CD20) and T-cells (CD3 and PD1) in human lymphoid (adenoid) tissue sections, see Fig. 1. The cellular localization was determined by a follicular dendritic cell marker CD35, specific for the B-cell compartment, the germinal center. For validation purposes, we showed that relative movement characteristics of the analyzed set of lymphocytes are comparable to lymphocyte movement calculated in two-photon microscopy experiments of transgenic mice^44^. For details on the extraction of movement-related parameters see the corresponding section in “Material and methods”.

**Figure 2:**
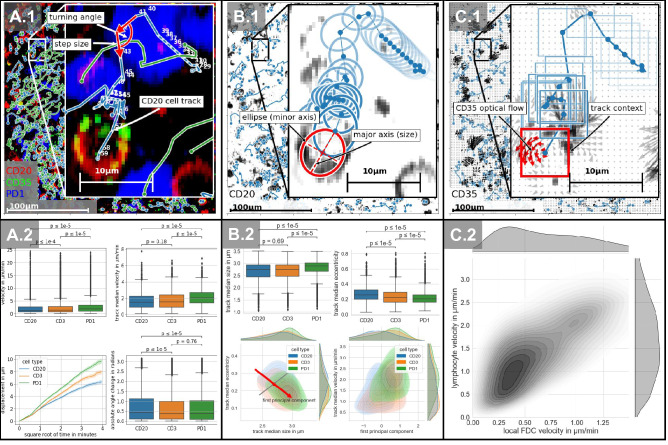
Movement and morphology of lymphocytes in human lymphoid tissue. Column A describes the analysis of the lymphocyte motility based on cell tracks. (**A.1**) visualizes the definition of the inspected features, turning angle and step size per time frame, later called velocity. (**A.2**) shows the results of the analysis. Column B describes the morphological analysis of lymphocytes based on cell tracks. (**B.1**) displays the features used for morphological descriptions, ellipse, to represent the eccentricity and major axis, later defined as cell size. On the upper left of (**B.2**), the size of each cell type is visualized. Conclusively, column C displays the analysis of CD35 positive follicular dendritic cells (FDC). (**C.1**) visualizes the major concept of optical flow to determine on spot FDC velocity. (**C.2**) shows the positive correlation of FDC velocity related to lymphocyte velocity in the microenvironment.

In order to evaluate the motility of lymphocytes, we investigated their velocity and turning angles on the basis of cell tracks, see Fig. 2A.2. At first, we measured the mean velocity per time frame as well as the average median velocity per track. Here, PD1 positive cells showed in both statistics significant higher velocities (Mean velocity: $2.55~ \upmu \text {m}/\text {min}$, mean track median velocity: $2.11~ \upmu \text {m}/\text {min}$) compared to CD20 (Mean velocity: $2~ \upmu \text {m}/\text {min}$, mean track median velocity: $1.65~ \upmu \text {m}/\text {min}$) and CD3 (Mean velocity: $2.06~ \upmu \text {m}/\text {min}$, mean track median velocity: $1.72~ \upmu \text {m}/\text {min}$) positive cells, shown in the upper row of Fig. 2A.2 . We consider differences as significant if the resulting p-value of the Mann–Whitney U rank test is below 0.0001, which is also indicated by horizontal bars above each barplot. Comparing the mean velocities of CD3 and CD20 positive cells, CD3 positive cells showed significantly faster movement, which is in accordance with mice experiments, even if the difference was not as pronounced^44^. We found no significant differences in median velocity between CD20 and CD3 positive cells. The approximated distribution of both velocity calculations followed heavy-tailed characteristics, which could be also shown in mice experiments^1^ (see Extended descriptive statistics of Supplementary materials). We were also able to confirm the random walk and diffusion process characteristics (Miller et al.^44^ and Parker et al.^15^) by showing the proportional increase of displacement over the square root of time (lower left of Fig. 2A.2). Beyond relative similarities, we measured on average slower absolute velocities in comparison to previous two-photon experiments of transgenic mice^44^ , see “Discussion” for an extended discussion. Concerning angle change during each track, CD3 as well as PD1 positive T-cells showed similar characteristics. CD20 positive B-cells had significantly higher alterations of direction compared to both sets of T-cells (lower right of Fig. 2A.2).

**Table 1:** The impact of temporal information on the classification of phenotypes.

(a) Information levels				
Level	Data	Model	Information content	
1	Track features	LR	Long-term movement	
2	Raw tracks	1d CNN	Short-term movement	
3	Raw 2D frames	2d CNN	Morphology	
4	Raw video patches	3d CNN	Morphology & movement	

(b) Intra-patient				
Level	CD3$\leftrightarrow$PD1	CD20$\leftrightarrow$CD3	Mean	
1	$0.65\pm 0.01$	$0.61\pm 0.06$	$0.63\pm 0.04$	
2	$0.53\pm 0.04$	$0.59\pm 0.02$	$0.56\pm 0.03$	
3	$0.93\pm 0.09$	$0.97\pm 0.04$	$0.95\pm 0.07$	
4	$0.92\pm 0.10$	$0.98\pm 0.02$	$0.95\pm 0.07$	
Mean	$0.76\pm 0.07$	$0.78\pm 0.04$		

(c) Inter-patient				
Level	CD3$\leftrightarrow$PD1	CD20$\leftrightarrow$CD3	CD20$\leftrightarrow$PD1	Mean
1	$0.64 \pm 0.03$	$\mathbf {0.61\pm 0.03}$	$0.67\pm 0.08$	$0.64\pm 0.05$
2	$0.61\pm 0.03$	$0.48\pm 0.02$	$0.65 \pm 0.09$	$0.58\pm 0.06$
3	$0.70\pm 0.03$	$0.51\pm 0.05$	$0.78 \pm 0.10$	$0.66\pm 0.07$
4	$0.75 \pm 0.04$	$0.50\pm 0.05$	$0.80\pm 0.05$	$0.68\pm 0.04$
Mean	$0.67 \pm 0.05$	$0.52\pm 0.03$	$0.72 \pm 0.08$	

Table (a) Information levels arising from different data representations that are used for training different models. Tables (b and c) show crossvalidation results reported as mean and standard deviation of the AUC out of 5 runs per fold, where for Table (b) results on 4 (CD3 PD1) and 5 (CD20 CD3) movies (leave-one-movie-out) and for Table (c) results on 24 movies with 3 folds (with no patient overlap) were used.

We evaluated the diameter as well as the eccentricity of B-cells and T-cells (Fig. 2B.2). PD1 positive cells showed the largest diameter whereas CD3 and CD20 positive cells were on the same scale. CD20 positive B-cells showed the most elongated morphology followed by CD3 positive T-cells. In order to investigate possible correlations between morphology and velocity, we summarized the variance of both diameter and eccentricity as the first principal component where we found that the eccentricity of a cell decreases with increasing size (see Fig. 2B.2 bottom left). Furthermore, we found that the velocity correlates with the first principal components, i.e. median eccentricity decreases with increasing median velocity whereas the median size increases. Although this seems contradictory to the amoeboid motion observed in previous experiments in mice^44^, we observed a correlation between the standard deviation of eccentricity and velocity, supporting the hypothesis of amoeboid motion.

Conclusively, we investigated the motility of follicular dendritic cells (FDCs) and possible implications on the motility of its microenvironment (Fig. 2C.2). The motion of the dendritic network, measured by optical flow, is localized and pulsative. We measured the movement of FDC in single image patches, where the size of the patch defines the size of considered context ($5~\upmu m$ here). Velocities of FDCs were around $\sim 0.27~\upmu \text {m/min}$. To investigate the impact of FDC movement on its direct environment, we only considered lymphocytes in the immediate vicinity of FDCs (closer than $8~\upmu \text {m}$). Occasionally CD35 can stain B-cells, which can usually be differentiated from FDCs by their round shape. There is a strong correlation between local FDC velocity and resulting contiguous lymphocyte velocity (Fig. 2C.2).

### The impact of temporal information on the classification of phenotypes

In the previous section, we identified slight statistical differences between phenotypes in terms of morphological and dynamic features. While such statistics are useful to draw inferences about populations from a sample, we are interested in patterns derived from a machine learning model to predict its phenotype. Before identifying and testing this biological hypothesis we evaluate which representation (movement or morphology alone, or the combination of them) gives us most discriminating power.

We performed an experiment to separate the different channels in the original videos and try to determine the phenotype from movement and/or morphology. This is based on different representations of the input data along with appropriate machine learning models to process them and refer to these combinations that increase in complexity and dimensionality as *information levels* (Table 1a). For each information level, we assess the ability to separate two phenotypes by framing the problem as a binary classification task, quantified based on the area under the receiver operating characteristic curve (AUC), where an AUC score of 1 denotes perfect separability. A random classifier would yield an AUC score of 0.5 (for more details about the representations, preprocessing, cell-tracking, models and its evaluation see “Material and methods”). In addition, we investigate the generalizability in two different settings, within a single patient and across patients.

The results of the experiment are shown for the intra-patient scenario Table 1b and for the inter-patient scenario in Table 1c. As a measure for the difficulty of the prediction task we also indicate the mean performance across all information levels. We find very high classification scores in the intra-patient scenario (best-performing methods at 0.93/0.98 AUC for CD3 vs. PD1 and CD20 vs. CD3, respectively). As expected, the intra-patient scenario that measures the generalization to unseen patients is more challenging and hence the corresponding classifications drop considerably compared to the intra-patient scenario (best-performing methods at 0.75/0.61/0.80 AUC for CD3 vs. PD1 and CD20 vs. CD3 and CD3 vs. PD1, respectively). Classification scores at least for CD3 vs. PD1 and CD20 vs. PD1 with best-performing scores of 0.75 AUC and 0.80 AUC, respectively, still represent a high degree of inter-patient generalization in a real-world biological system.

The general inspection of individual information levels reveals a largely consistent ranking. We see that overall morphology-based classifiers (level 3) and classifiers based on movement and morphology (level 4) outperform movement-based classifiers (level 1 and 2). The intra-patient classification based on the information level 4 showed outstanding AUC scores (Average AUC: 0.95), but the level 3 classifier performs equally well in terms of mean AUC score. This entails that for single patients, movement information gives barely measurable advantages for the definition of cell entities. The advantage of temporal context gets more pronounced in the inter-patient scenario. We measured better classification scores for level 4 in comparison to level 3 based classifications that, however, still remain consistent with each other within error bars. Nevertheless, both in the case of CD3 vs. PD1 and CD20 vs. PD1, there is a consistent trend in favor of the level 4 classifier, which relies on both movement and morphology.

Cell-type-specific investigations showed that overall PD1 positive follicular T-helper-cells are best distinguished from T-cells and B-cells (Average AUC: 0.72). Here, we see clear differences in morphology and movement, intra-patient (level 4, Average AUC: 0.92) as well as inter-patient (level 4, Average AUC: 0.78), for activated follicular T-helper-cells. Regarding the discrimination of T-cells and B-cells (CD3 vs. CD20), we obtain the best score for the intra-patient classification (Average AUC: 0.79, level 4, Average AUC: 0.98). So inside a single patient T-cells and B-cells are clearly distinguishable.

This result is in strong contrast to the performance level for the same task reached in the inter-patient scenario, where none of the classifiers apart from information level 1 are discriminative in the sense that all other AUC scores remain consistent with the non-discriminative value 0.5 within error bars. The best classification score is reached for information level 1, which builds on general movement characteristics of T-cells and B-cells (Average AUC: 0.61). Even though level 2 and level 4 both take movement information into account, the level 4 model is not able to distinguish between B and T cells (which is caused by smaller temporal context due to architectural limitations).

### In silico prototypes of lymphocytes derived from machine learning models

While the descriptive, statistical analyzes facilitates rule-based modelling on few, manually selected properties, machine learning approaches integrate a large set of properties and their context. Visualizing the most predictive samples provides a way of understanding prototypical patterns that have been identified by the machine learning model. For models based on morphology we superimpose these input-space visualization by interpretability heatmaps obtained from layer-wise relevance propagation (LRP, see^45,46^), which attribute in the input domain how much specific pixels contributed towards the classification decision.

#### Morphological analysis

**Figure 3:**
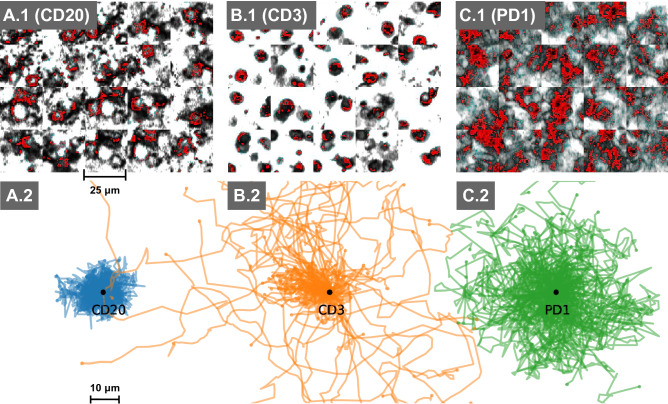
In silico prototypes of cell entities and its environment based on supervised machine learning models. Here, prototypes are defined as most predictive samples from inter-patient experiments. The top row visualizes the morphological and environmental prototypes of cell entity derived from convolutional neural networks. Red pixel highlight most predictive pixels computed by LRP analysis of the respective discriminative machine learning model. The bottom row displays the most typical tracks per cell type based on Logistic Regression Analysis.

We inspected the top true positive patches out of the inter-patient analysis of data level 3 and the top true positive tracks of data level 1. Figure 3A.1–C.1 depicts single frames of isolated channels from the individual cell entities. Grey pixels represent positive pixels of the individual immunohistological staining. Red pixels represent cell entity specific pixel output of the LRP analysis of the corresponding convolutional neural network, highlighting the areas which are important for the prediction.

Follicular T-helper-cells tend to form undefined morphologies with blurred intermediate intensities inside a dense microenvironment (Fig. 3C.1). In contrast, T-cells show a small round morphology with clearly defined and densely stained surfaces located inside a sparse microenvironment (Fig. 3B.1). B-cells tend to build highly connected surfaces with clearly defined cell bodies surrounded by other B-cells (Fig. 3A.1).

Our cell-specific qualitative description aligns well with the underlying pixel attributions computed with LRP analysis. This type of analysis allows for generating insights on the one hand, but also to build trust in the model on the other hand (i.e. ruling out the existence of *Clever Hans effects* caused by artifacts such as noise and other spurious correlations^46^).

We analyzed the models perspective on data level 1 (top true positives), shown in the bottom row of Fig. 3A.2–C.2. This analysis shows specific long-term movement behaviour of each cell entity according to a supervised model. B-cells tend to perform a local movement, characterised by a small rate of displacement and large turning angles in each step (Fig. 3A.2). In contrast, T-cells seem to move in more directed way, characterised by a high displacement and a small deviation of turning angles (Fig. 3B.2). Follicular T-helper-cells are defined by high displacements and a high standard deviation of turning angles (Fig. 3C.2). The different movement of T- and B-cells has been examined in mouse model^1^, stating a relative higher velocity for T-cells similar to our results.

Those conclusions are not possible to draw by Fig. 2A.2 alone. While simple statistics allow for rough observations in general (recall from Fig. 2A.2, where e.g. CD20 and CD3 show comparable velocities while at the same time having distinct distributions of angular changes), analysis of supervised trained classification models allow for better identification of more specific distinct characteristics. In the descriptive statistics of Supplementary Material, we extend the analysis by highlighting differences between inter and intra patients experiments (see Fig. 5 of Supplementary material). Moreover, in Fig. 6 we analyzed error rates and associated patterns within in each cell type (for both inter and intra patient) uncovering further insights in the mechanisms of convolutional neural networks.

### The definition of motion-specific subsets of cell types

Here, we collect further evidence supporting the role of movement as a complementary ordering principle, which can be used to identify subpopulations both within and across different CD-clusters.

In Figs. 2 and 3, we show that cell entities defined by CD clusters generally exhibit specific patterns in terms of both movement and morphology. Especially, activated follicular T helper cells (PD1) show large diameters and rapid movement. T-cells and B-cells also show specific morphological and movement patterns in individual patients. However, the movement- and morphology-based comparison of T-cells and B-cells across patients remains a challenge.

Comparing long-term movement patterns of T-cells (CD3) and B-cells (CD20) (Fig. 3), T-cells show on average higher displacements and lower standard deviations of turning angles in comparison to B-cells (Fig. 2A.2). T-cells shows significantly lower absolute angles changes as compared to B-cells, while there was no significant difference in terms of velocities. Track features (level 1, see 1a) were the only level of representation that allowed to distinguish both cell types (at least partially) across patients (Table 1c).

**Figure 4:**
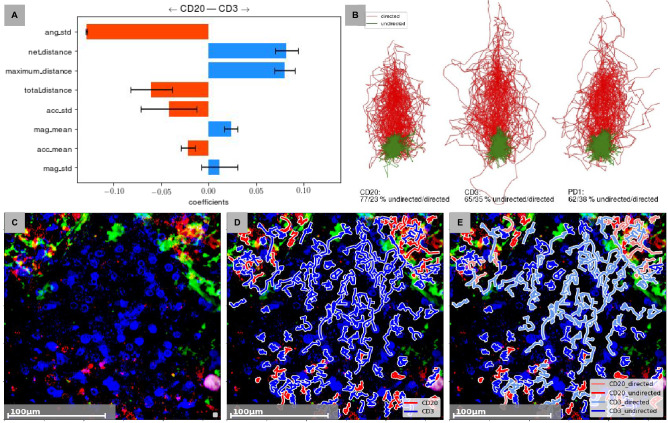
The definition of motion-specific subsets of cell types. The so called data-driven clusters (DD-cluster), directed and undirected, are based on eight most discriminative features derived from binary logistic regression experiment CD20 vs CD3, shown in (**A**). (**B**) displays the composition of DD-clusters per cell type, defined by unsupervised machine learning. Conclusively, (**C**–**E**) visualize the data enrichment based on movement and machine learning. (**C**) shows information given by conventional immunohistochemistry. (**D**) visualizes the information given by immunohistochemistry enriched with movement and computer vision. (**E**) visualizes information facilitating immunohistochemistry, movement, computer vision and machine learning.

We use unsupervised clustering methods to identify movement-specific clusters, so called data-driven clusters (DD cluster). For this we first compute the two most descriptive features resulting from the inter-patient logistic regression of T-cell and B-cell populations, especially the displacement and the standard deviation of turning angles per track (as can be seen in the Fig. 4A). Applying k-means clustering to these features resulted in two bipartite sets of cells showing directed, as well as undirected movement patterns (Fig. 4B). Here, cell tracks with a directed movement are colored in red and cell tracks with an undirected movement are colored in green. It can be seen that T-cells, B-cells and follicular T-helper-cells enclose cells of both DD-clusters. However, the composition per DD-clusters differs for the single CD-cluster-based cell entities. B-cells contains the largest proportion of cells ($77\%$ undirected, $23\%$ directed) that show undirected movement. In comparison we see less undirected moving cells in the T-cell population ($65\%$ undirected, $35\%$ directed). The highest amount of cells showing undirected movement patterns are detected for the follicular T-helper-cell population ($62\%$ undirected, $38\%$ directed).

The DD-clusters are exclusively defined by the long-term movement behaviour of cells, allowing for a more fine-grained characterization of sub-populations within the given framework of CD-clusters. Further analysis of the cluster revealed no significant differences in morphology suggesting the necessity for temporal information. This approach is illustrated in the lower row of Fig. 4, where C is the raw frame, D shows again the result of standard cell tracking and in E we colored the tracks according to their cluster membership (pale color for directed tracks).

## Discussion

The function of a cell can be reflected in its morphology and movement^44^. We addressed the idea to extrapolate from 3D and 4D information to cellular function and behaviour with machine learning. In the beginning, we evaluated morphology and movement of T-, B- and dendritic cells in human adenoid tissue in the context of well-defined mouse experiments. Here, the evaluation of cellular motility and morphology revealed comparable rankings of velocity and angular change. Beside comparable relative statistics, we measured lower absolute velocities and displacements of lymphocytes.

Miller et al.^44^ examined the motion of lymphocytes inside lymph nodes of transgenic mice via two-photon microscopy. In comparison, we examined the motion of lymphocytes inside human adenoids via confocal microscopy. Using tracks provided by proprietary software [Imaris Advanced Tracking 9.5 from Bitplane AG (Badenerstraße 682, CH-8048 Zurich, Switzerland)] and extracting velocities via forward derivatives leads to values that are comparable with values reported in the mice literature (mean track median velocity: CD20: $6.36~ \upmu \text {m}/\text {min}$ , CD3: $6.57~ \upmu \text {m}/\text {min}$, PD1: $8.13~ \upmu \text {m}/\text {min}$). These tracks do not always contain consecutive time steps and require appropriate imputation, which leads to a reduction of about 15% in terms of absolute velocities. For increased numerical stability, we used centered differences to infer velocities from track positions. Using simple forward derivatives would lead to an increase of about 35% for the reported velocities in the results section. For the later analysis of the raw movies with CNNs, we applied blurring during preprocessing and used the same setup also for the descriptive statistics for reasons of consistency. The omission of this step would amount to an increase of the measured velocities of approximately 50%. In addition, there is a application-specific selection bias in the different tracking software packages. The proprietary software tends to return many short tracks that lead to an increase of the mean velocity. Finally, the reported absolute velocities were measured in a two-dimensional maximum intensity projection. The three-dimensional tracks obtained from the proprietary software allow to assess the impact of this simplification and lead to an approximate decrease of 17% when comparing velocities in three-dimensional space to absolute projected velocities. Taking into account the agglomeration of all the effects mentioned above, the reported velocities are comparable to values reported in the mice literature.

Beside lymphocyte movement based on cell tracks, we analyzed the motility of CD35 positive FDC based on optical flow and its implications for its microenvironment. We were able to reveal a positive correlation of FDC and lymphocyte velocity. Furthermore, the visual examination of FDC motion indicates a possible correlation of specific movement patterns of FDCs and the behaviour and function of lymphocytes in the microenvironment, which has to be proven in upcoming studies. Here, we identified an undirected, pulsative motion of the network during possible probing processes of surrounding lymphocytes. In addition, we saw directed stretches of the flexible network morphology when lymphocytes moved directly on top of the network.

In the statistical analysis, we already found slight differences between different phenotypes in terms of morphological and/or movement-related parameters. To evaluate those findings and the gain of information, we performed an extensive analysis using different information levels, see Table 1a. In intra-patient context, the binary classification performed equally on morphology data only and with added movement data. Considering inter-patient data, the combination of morphology and movement data outperformed the morphology only approach, slightly. In the same context, PD1 positive T-helper cells were best distinguishable from both other cell types, i.e. CD3 positive T-cells and CD20 positive B-cells. This observation is explainable as PD1 positive cells may show high activation level. Inside a single patient T-cells and B-cells are clearly distinguishable.

In the inter-patient context, the classification performs best on track feature data (level 1). At first sight, it might seem contradictory that neither level 2 nor level 4 can identify pattern that generalize across patients even though both of them also have access to movement information. We investigated this apparent paradox by adjusting the maximum track length (Fig. 3 in Supplementary material), revealing, on the one hand, that the performance quickly degrades with reduced temporal context size (track length). This explains the weak performance of the level 2 classifier as a shallow CNN with a correspondingly very limited receptive field, which exploits mostly local short time movement characteristics. On the other hand, the classification performance begins to saturate at 60 timeframes and hence is not expected to increase drastically for even larger temporal context, which provides a post-hoc justification for limiting the recording time to 20 minutes. The failure of morphology-based and/or short-term-movement-based classifiers can most likely be attributed to the fact that the morphology and in-time movement characteristics are highly dependent on the lymphoid conditions. The high variance is consistent with the broad range of reactive morphological patterns of B-cells and T-cells, stated in the literature^3,16,24,44^. In contrast, long-term movement patterns turned out to provide at least a stable, discriminative signal, which generalizes to a certain degree across patients. Summarizing previous findings, it seems that including movement characteristics into scientific workflows enables to solve specific challenges, insoluble by current state of the art techniques. However, it was also shown that the associated additional effort and complexity does not correlate with an increase in information for every scientific task.

**Figure 5:**
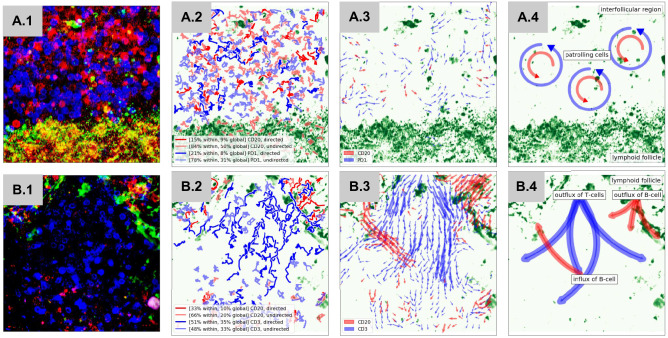
Functional information hidden in the context of cell movement. The rows A and B show the full evaluation of two videos, including traditional 2D morphology and the new movement characteristics. (**A.1**) shows a dense region near follicular dendritic cells filled with B cells and PD1 positive cells. (**B.1**) represents a sparse region mainly filled with T cells. The morphologies of the present cells are round and compact. Inspecting tracks and DD clusters of B and T cells inside both regions (**A.2** and **B.2**), we can see an over representation of long and directed tracks inside (**B.2**). compared to (**A.2**). The over representation of directed DD clusters indicate a more active region. These hints can be confirmed by the collective behaviour of B cells, T cells and PD1 positive cells. We visualized the optical flow of the last 30 frames of Video A (**A.3**) and B (**B.3**). A.3 reveals a localized circular movement pattern indicating patrolling cells inside an interfollicular region, schematically show in figure (**A.4**). (**B.3**) reveals an in and outflux of T cells and B cells from a lymphoid follicle, schematically shown in figure (**B.4**). Here, we can show, that DD clusters and collective movement patterns has the possibility to reveal cellular states and functions, which can not be detected by the evaluation of cell morphologies inside static images.

Figure 4 visualizes the DD-clusters calculated based on the long-term movement characteristics, indicating different movement pattern within the cell groups defined by CD clusters. Here, we go one step further by facilitating movement information and machine learning. In the context of cellular function, directed movement implies a guidance of cells, whereas an undirected and circular movement can reflect an on spot patrolling or probing of cells (Fig. 5). Cells can be guided under different circumstances such as a previous probing of an antigen-presenting cell or different chemokines. Therefore, the correlation of movement and function implies an indirect differentiation of functional subgroups.

## Conclusion

In this work, we demonstrated the significance of cellular motion for a comprehensive and generalized definition and understanding of human lymphocytes (Fig. 5). By combining machine learning with a unique 4D dataset of fresh human adenoid tissue sections we could—for the first time, to the best of our knowledge—investigate the behavior of human lymphocytes at different temporal scales and measure the information content of different pathomic abstraction levels.

First, we provided a quantitative assessment of kinematic and morphological parameters of lymphocytes in human lymphoid tissue revealing distinct characteristics of the different cell types (Table 2). In addition, we validated the 4D dataset in the context of mice experiments.

**Table 2:** Short qualitative description of considered cell types in terms of most likely (prototypical) properties given at different levels of abstraction.

Level	Property	CD20	CD3	PD1
Track	Velocity	Slow	Slow	Fast
Track	Step angle	High	Low	Medium
Track	Size	Small	Small	Large
Track	Ecc	High	Low	Lowest
Track	Displacement	Low	High	Medium
Patch	Micro-env	Dense	Sparse	Diffuse
Patch	Morphology	Connected	Small/round	Undefined
Cluster	Ratio directed	Low	Medium	Medium

To measure the importance of temporal context for histological analysis in general, we proposed a supervised classification schema as a proxy for quantifying the impact of temporal context. Furthermore, the expressiveness of the respective representation was studied by highlighting the advantages in terms of model analysis for each of the proposed representation. In particular, we showed that inferring the phenotype from movement and/or morphology is possible in most cases, to some extent even across patients. Moreover, we contributed prototypes of cell types comprising morphology, microenvironment and motility, that provide additional morphological insights compared to static histopathological images, see patch-level entries in Table 2. These findings corroborate the hypothesis that the additional temporal information allows for a more precise understanding of the human lymphoid tissue. We were able to group cells based on their movement across different cell types. These clusters were identified based on global movement characteristics and can be broadly characterized as undirected vs. directed motion. This analysis allows for a more specific description of cell entities going beyond conventional antibody staining. Currently cell types are exclusively defined by CD-clusters, but future investigations will result in an expanded functional differentiation of the heterogeneous set of lymphocytes. Moreover, future work should aim at the functional validation of DD-clusters as well as pathomic changes comprising morphology and movement in different pathologies.

## Material and methods

### Data preparation

The dataset consists of tissue samples of fresh human adenoids of 10 patients. The tissue originates from a group of patients comprising young people showing a pharyngeal tonsil hyperplasia. The samples were obtained via routine adenectomy by the ear-nose-throat center of the University Hospital Frankfurt am Main. Informed consent of all patients was obtained in accordance with the Declaration of Helsinki. The local ethics committee of the University Hospital Frankfurt (No. 387/16) approved this study. All tissue samples were anonymized and processed in accordance with the institutional guidelines of the Johann-Wolfgang-Goethe-University/Frankfurt and cannot be traced back to an individual person. The protocol of Donnadieu et al.^21,22^ was applied. The fresh adenoid specimens were embedded in a 5% low-melting-temperature agarose and cut into $350~\upmu \text {m}$ thick sections with a vibratome. The fresh slices were stained for 15 min at $37^\circ \text {C}$ with solutes diluted in RPMI 1 without Phenol red with a concentration of $10~\upmu g/ml$. For the visualization of T-lymphocytes, B-lymphocytes and FDC, we used the following Abs: Alexa Fluor 647-anti-human PD-1 (clone EH12.2H7; BioLegend), FITC-anti-human CD35 (clone E11; BioLegend), Alexa Fluor 647-anti-human CD3 (clone UCHT1; BD Biosciences), Brilliant Violet 421-anti-human CD3 (clone UCHT1; Biolegend), and Pacific Blue-anti-human CD20 (clone 2H7; BioLegend). A Leica SP8 confocal microscope (Leica Microsystems, Wetzlar) and a corresponding thermostated chamber for livescans were used. The experiments were conducted under controlled conditions for oxygen and temperature (constant $37^\circ \text {C}$). During the scanning phase, the specimen were perfused at a rate of 0.8 *ml*/*min* with a solution of RPMI 1640, bubbled with $95\%~O_{2}$ and $5\%~CO_{2}$. 24 video sequences with three different stain combinations were obtained: 11 (4 patients with 3 + 2 + 1 + 1 videos) $\times \mathrm {(CD3,CD35,PD1)}$, 7 (4 patients with 4 + 3 + 3 + 1 videos) $\times \mathrm {(CD20,CD35,PD1)}$ and 6 (2 patients with 5 + 1 videos)$\times \mathrm {(CD20,CD35,CD3)}$. Part of the data were published in another context and applying different methods^22^. The total scanned horizontal areas ranging from to $32-68~{\mathrm {mm}}^2$ with different spatial resolutions ranging from 0.1 to $0.4~\upmu \mathrm {m}/\mathrm {pixel}$. The temporal duration ranging from 14-21 minutes keeping the same temporal resolution ($\approx$ 20 s).

In order to reduce data heterogeneity, several selection criteria were imposed: (1) passed initial quality control i.e. no fluid artefacts or excessive noise (2) all three channels populated homogeneous, in particular enough CD35 (no completely black areas) (3) total size (after scaling to common resolution) between 400 and 600 pixels (4) elapsed time between sampling and recording s.t. cells are alive and (5) homogeneous staining (no obvious decrease of fluorescence with time).

### Preprocessing

These video sequences were downscaled and normalized along the spatial and temporal dimensions. Thus, they share the same spatial ($\approx 0.4$ μm/pixel) and temporal ($\approx$ 20 s) resolution. For further processing of the data, we decided to calculate the maximum intensity along the z-stack, so that the resulting dataset represents a sequence of 2D images. This allowed us to build upon well-founded knowledge from computer vision. The last preprocessing step involved color equalization and contrast adjustments. In all the experimental results, the cells’ velocity magnitude and direction are reported in μm/min (using 1.23 as a conversion factor) and *radians*, respectively. An overview of the preprocessed dataset is shown in Fig. 1E each composed of three channels (A–C). Contrast was adjusted (low intensity cut-off, blurring) and normalized such that noise and contrast level across all videos became equal.

### Cell tracking and feature extraction

For each channel $\in \{\mathrm {CD20},\mathrm {CD3},\mathrm {PD1}\}$ we extracted cell tracks using *trackpy*^47^. For reasons of reproducibility, we decided to use the open source cell tracking package trackpy instead of tracks from the proprietary Imaris software. In total, this resulted in 7929 tracked cells, where 2476, 2805 and 2648 were tracked for CD20, CD3 and PD1 respectively. To guide the tracking into more reasonable solutions, we found following parameters (in terms of pixels) to be useful: $\textit{cell diameter}=25$, $\textit{separation}=15$, $\textit{maximum speed}=20$ (as an upper bound), $\textit{memory}=10$ (for connecting fragmented tracks), $\textit{filter\_frames}=20$ (as minimum track length) and $\textit{minmass}=5000$ (as the sum of raw pixel intensities stored as bytes).

Each track of length *l* is then represented as a series of triples $x_i = \left[ (t_0,x_0,y_0),\ldots ,(t_l,x_l,y_l)\right]$, where *t* is the time point at which this point appear in the video, and *x*, *y* are the respective locations. We then first represented each track relative in terms of step size (velocity), change in step size (acceleration) and turning angles, where velocity and acceleration was determined by computing the euclidean norm of the first and second derivative respectively. Turning angles are defined as the change in direction between two consecutive segments reported as radians (i.e. in the interval between $\left[ -\pi ,\pi \right]$, where positive and negative angles corresponds to clockwise and anti-clockwise turning angles). Thus, each track of length *l* in then represented as a series of triples $\hat{x_i}=\left[ (v_0,a_0,r_0),\ldots ,(v_l,a_l,r_l)\right]$, where *v* is the step size, *a* change in step size and *r* the turning angle. This type of representation ensures invariance with respect to translation and rotation, a property which is desired when analyzing cell dynamics.

For our feature based (logistic regression) model we also computed multiple statistics for each column of our representation, namely the mean, standard deviation, minimum and maximum value. In addition, we enriched our feature set with three different distances based on each $x_i$: (1) $\textit{total\_distance}$ as the integral of all step sizes, (2) $\textit{net\_distance}$ as the distance between start and endpoint (i.e. $\Vert x_{il} - x_{i0} \Vert$) and (3) $\textit{maximum\_distance}$ as the maximum distance between start and any point of the track (i.e. $\text {max}(\left\{ \Vert x_{il} - x_{it} \Vert \text {for all } 0< t < l \right\} )$.

### Representations and machine learning models

Since we want to study the influence of different representations (movement vs morphology) with respect to a downstream task, we aimed for a disentangled experimental design allowing for drawing conclusions based on performance. For this, our proposed methods are of general relevance for the characterization/analysis of moving cell data and can be divided into three major groups:

1. *Movement only data*: where the data ${\mathbf {X}} \in {\mathbb {R}}^{N\times L \times 3}$ consisting of *N* tracks each witch different length *L* (ranging from 20 to 60) where for each triple $x_i \in {\mathbb {R}}^{L \times 3}$, $(v_i, a_i, r_i)$ is given as described above. This type of representation is further divided into two groups, where we fit models based on
  - raw track data with 1D convolutional neural networks (1DCNN), where the input sequences are padded to a common length. The network architecture consists of three layers each consisting convolutional filter banks with 128,64,32 filters each with size 3 and ReLU activations followed by a max pooling layer of size 2. After those three layers, a global maximum and average pooling is applied and concatenated before the our classification head (with softmax activation). To prevent overfitting on training data we regularized the weights with L2-regularization (.001) and applied Dropout. The model is then optimized with standard stochastic gradient descent (with learning rate .001) minimizing crossentropy.
  - a feature based logistic regression model, where each sample is a 15 dimensional (3 (velocity, acceleration, angle change) × 4 (mean, std, min, max) + 3 (total, net and maximum distance)) vector consisting of standardized features (as described above), i.e. features that have mean zero and variance one.
  Apparently these models have only access to the extracted movement data without any knowledge about morphology. In this study we refer to morphology as all possible information which can be derived from an image by a convolutional neural networks, this includes size and shape of cells, but also textures and features from the microenvironment.
2. *Morphology only data*: where the data ${\mathbf {X}}\in {\mathbb {R}}^{N\times M \times X \times Y}$ consisting of *N* movies times *M* frames, where each frame has $X \times Y$ pixels. Since only random patches (of size $64 \times 64$) from single frame are fed to a standard 2D convolutional neural network (2DCNN), this type of model has only access to morphological data apparent in one frame. This corresponds to the traditional setup of applications of neural networks to histopathological problems operating on single whole slice images. The network architecture also has three layers each consisting of 256, 128, 64 filters each of size $3 \times 3$ followed by max pooling of size 2. Theses layers are again followed by maximum and average global pooling, concatenation and a shallow classification head. In contrast to 1DCNN, our 2DCNN (and also 3DCNN) are optimized using Adam^48^ with learning rate 0.001. Again we used L2 weight regularization and dropout to prevent overfitting.
3. *Movement and morphology data*: where ${\mathbf {X}} \in {\mathbb {R}}^{N \times M \times X \times Y}$ consisting of *N* movies each with *M* frames, where each frame has $X \times Y$ pixels. The architecture is differs only in the filter size, namely $3\times 3 \times 3$, and the input size ($16 \times 64 \times 64$). Since this setup is fed with consecutive frames, it becomes possible for the model to exploit temporal dependencies useful for the downstream task.

Although we propose and evaluate only one specific choice of architectures and input sizes, we also experimented with different architecture and especially with different spatio temporal patch sizes. These experiments suggested our proposed choice and was mainly driven by a trade-off between model complexity and downstream performance. Although higher spatial context increased performance for both (2D and 3DCNN), we went for patch size of 64 to keep both (2D and 3DCNN) comparable i.e. identical up to the temporal context size.

### Experimental design and evaluation

In order to compare performances for different representations with different model architectures in the context of discriminating between different phenotypes (CD20, CD3, PD1), we decided to focus on three binary classifications tasks instead of one classification of three classes. This experimental design allows for better identification of problems associated with each representation and the corresponding task. In case of intra-patient experiments, we divided into three folds, in case of single patient we computed the whole leave-one-movie-out-cross-validation. To account for slightly different results caused by random initialization, each fold was fitted five times. We report the overall mean area under the ROC curve (AUC).

In order to allow for comparability we evaluated all the methods the same way s.t. each method returns predictions for each pixel in the videos. In case of spatial models on raw movie input this is achieved by applying the model to each spatio-temporal point in the video. In order to avoid border-artefacts we applied overlapping moving windows. In case of movement-based models (1D-CNN on raw tracks or Logistic Regression on precomputed features) applied the predictions for each track to the corresponding spatio-temporal pixels with some width around. Filling the gaps (i.e. areas where there are no tracks) is achieved via postprocessing the prediction with watershed algorithm, i.e. each blank pixels is assigned to the nearest predicted spatio-temporal pixel. This type of evaluation has several advantages, where the most prominent is the fact that it allows for comparison across very different types of representations and model architectures. But also this increases the resolution available for evaluation (since evaluating on whole movie statistics yield poor statistics because of low sample size), avoid evaluating empty areas with no cell context.

### Techniques for interpretation of machine learning models

In principle we proposed two models: (1) a logistic regression model which is interpretable by design, i.e. the sign and magnitude of coefficients (weight matrix) is directly related to the input feature. And (2) neural networks (1D-,2D- and 3DCNN) which are highly non-linear models for very high dimensional and hard to interpret in terms of input patterns. Here, we used propagation-based methods, in particular layer-wise relevance propagation (LRP)^45,46^. This combination of models and attribution method has already been used for action recognition in video sequences^49^.

## Supplementary Information

Supplementary Information.

Supplementary Legends.

Supplementary Figure 4.

Supplementary Figure 4.

Supplementary Figure 4.

Supplementary Figure 4.

Supplementary Figure 4.

Supplementary Figure 4.

## Data Availability

The datasets generated and/or analyzed during the current study are available in the zenodo repository https://doi.org/10.5281/zenodo.5897619.
