# Supplementary material for: New definitions of human lymphoid and follicular cell entities in lymphatic tissue by machine learning
Source: Sci Rep. 2022 Nov 8;12:18991. doi: 10.1038/s41598-022-18097-9 (PMC9643435; doi:10.1038/s41598-022-18097-9)
Supplement: Supplementary file 1 — Supplementary Information. [file 41598_2022_18097_MOESM1_ESM.pdf]

New definitions of human lymphoid and follicular cell entities in lymphatic tissue by machine learning — Supplementary Information —

Patrick Wagner[a,b], Nils Strodthoff[a,c], Patrick Wurzel[d,g,m], Arturo Marban[a,b], Sonja Scharf[d,g,m], Hendrik Schäfer[j], Philipp Seegerer[b], Andreas Loth[n], Sylvia Hartmann[l], Frederick Klauschen[h,i,j,k,*], Klaus-Robert Müller[b,e,f,i,*], Wojciech Samek[a,i,*], and Martin-Leo Hansmann[d,m,*]

[a]Fraunhofer Heinrich Hertz Institute, Berlin, Germany
[b]TU Berlin, Berlin, Germany
[c]Oldenburg University, Oldenburg, Germany
[d]Frankfurt Institute for Advanced Studies, Frankfurt, Germany
[e]Korea University, Seoul, South Korea
[f]Max-Planck-Institut für Informatik, Saarbrücken, Germany
[g]Goethe University Frankfurt, Molecular Bioinformatics, Frankfurt, Germany
[h]Ludwig-Maximilians-Universität, Munich, Germany
[i]BIFOLD – Berlin Institute for the Foundations of Learning and Data, Berlin, Germany
[j]Charité - Universitätsmedizin, Berlin, Germany
[k]German Cancer Consortium (DKTK), Munich partner site, and German Cancer Research Center (DKFZ) Heidelberg, Germany
[l]Senckenberg Institute of Pathology, Goethe University, Frankfurt, Germany
[m]Institute for Pharmacology and Toxicology, Goethe University, Frankfurt, Germany
[n]Department of Otorhinolaryngology and Head and Neck Surgery, Goethe University, Frankfurt, Germany
[*]Correspondence to FK, KRM, WS and MLH.

ABSTRACT

Histological sections of the lymphatic system are usually the basis of static (2D) morphological investigations. Here, we performed a dynamic (4D) analysis of human reactive lymphoid tissue using confocal fluorescent laser microscopy in combination with machine learning. Based on tracks for T-cells (CD3), B-cells (CD20), follicular T-helper cells (PD1) and optical flow of Follicular dendritic cells (CD35), we put forward the first quantitative analysis of movement-related and morphological parameters within human lymphoid tissue. We identified correlations of Follicular dendritic cell movement and the behavior of lymphocytes in the microenvironment. In addition, we investigated the value of movement and/or morphological parameters for a precise definition of cell types (CD clusters). CD-clusters could be determined based on movement and/or morphology. Differentiating between CD3- and CD20 positive cells is most challenging and long term-movement characteristics are indispensable. We propose morphological and movement-related prototypes of cell entities applying machine learning models. Finally, we define beyond CD clusters new subgroups within lymphocyte entities based on long term movement characteristics. In conclusion, we showed that the combination of 4D imaging and machine learning is able to define characteristics of lymphocytes not visible in 2D histology.

1 Extended descriptive statistics

Here, we extend our analysis from the results section of the main paper (Insights from descriptive statistics). While we decided to focus on the quantitative differences between different cell types with respect to their movement, we focused on a qualitative description of the observed empirical distributions (see see Table 1a for simple statistics). To this end, we investigated multiple parametric distributions fits and selected according to Kolmogorov-Smirnov-Tests.

Figure 1 describes the movement of PD1, CD3, and CD20 cells in terms of velocity and angular changes, obtained from tracking data. The distribution illustrated in Figure 1C can be decomposed into two marginal distributions (for each cell type). The first marginal distribution, shown in Figure 1A, models the cells' velocity magnitude and corresponds to a log-normal distribution (best fit among log-normal, exponential and Levy-distributions). On the one hand, the data suggests that most

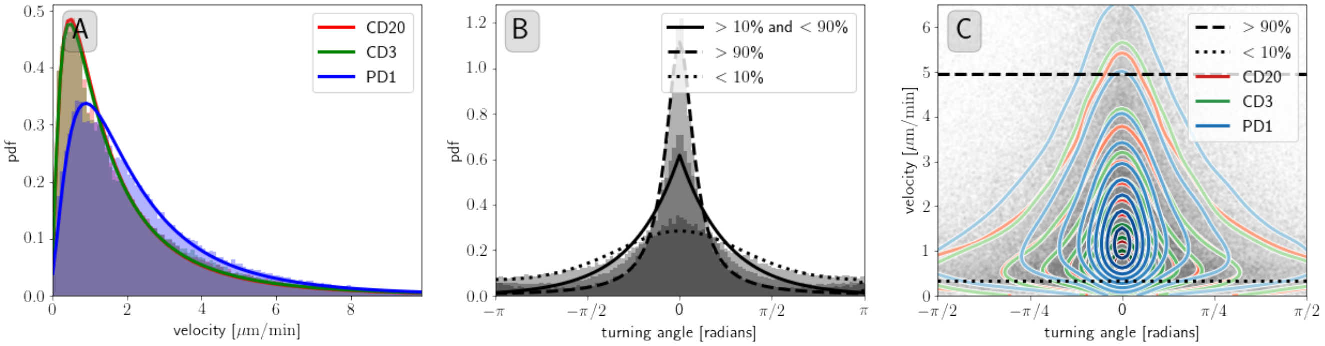

Figure 1. Extended descriptive analysis of velocity and angular distributions of. A: Distribution of velocities according to the cell type (CD3, CD20, PD1). The histogram bars correspond to empirical measurements, while solid lines to the best fit of the log-normal distribution. B: Distributions of cells' turning angles for different velocity percentiles computed from the 2D distribution shown in sub-figure (C). The dashed line describes a Cauchy distribution, which models cells' turning angles restricted to velocities greater than the 90 % percentile. Likewise, the dotted line corresponds to a von-Mises distribution and represents the best fit to cell velocities below the 10 % percentile. Finally, the solid line is a Laplace distribution and models cell velocities in the remaining interval, that is, between 10 % and 90 % percentiles. C: Two-dimensional joint distribution of cells' turning angle vs. cell velocity (for each cell type). The 10 % and 90 % percentiles described in sub-figure (B) are illustrated in this plot with the dotted and dashed lines (in black color), respectively.

cells prefer to move at lower speeds (i.e., the probability peaks at approximately $1\mu m/min$). On the other hand, the chance to find cells moving at high speeds is low (i.e., the probability decreases almost exponentially for cell speeds beyond $1\mu m/min$). The second marginal distribution, shown in Figure 1B, describes the cells' turning angle (i.e., the relative angle between the current and previous time step). Due to its complexity, it is modeled in three intervals, defined by the 10% and 90% percentiles computed along the velocity magnitude dimension (see the horizontal dotted and dashed lines in Figure 1C). The cells' turning angles above the 90% percentile are best described by a Cauchy distribution and those below the 10% percentile by a von-Mises distribution. The remaining interval, between 10% and 90% percentiles, is best modeled by a Laplace distribution. This data reveals that most cells prefer to keep their current direction while moving (i.e., the probability peaks at 0 radians). In contrast, the chance to find cells that change their direction of motion remains low (i.e., the probability decreases as the turning angle goes away from 0 radians). Moreover, as the velocity increases, cells are less likely to change their direction of motion (i.e., the Cauchy distribution, which models cell velocities above the 90% percentile, is more narrow and sharp at 0 radians than the other distributions. This behavior is well studied in many areas of life sciences and is expected as a consequence of conservation of angular momentum from physics.

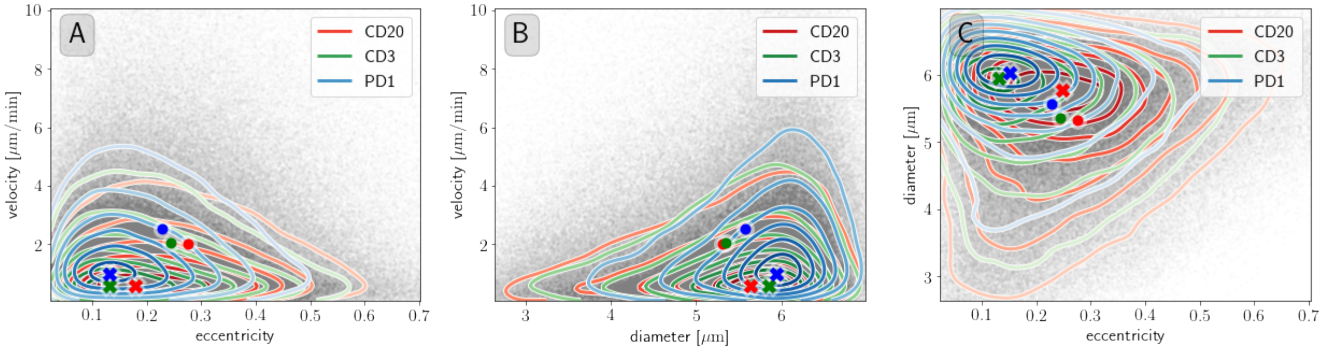

Figure 2. Descriptive analysis of cell-specific parameters as obtained from the tracking data. Raw measurements are scattered as gray dots, contour levels (color coded by cell-type) are kernel density estimations of the underlying respective subset. In addition we provide maximum likelihood estimate of the kernel and median of data as bold crosses and dots in respective colors. A: Velocity vs. eccentricity. B: Velocity vs. diameter (diameter of gyration of its Gaussian-like profile). C: Diameter vs. eccentricity.

Figure 2 illustrates a series of 2d joint distributions obtained from the tracking data (note that these plots differ from Figure

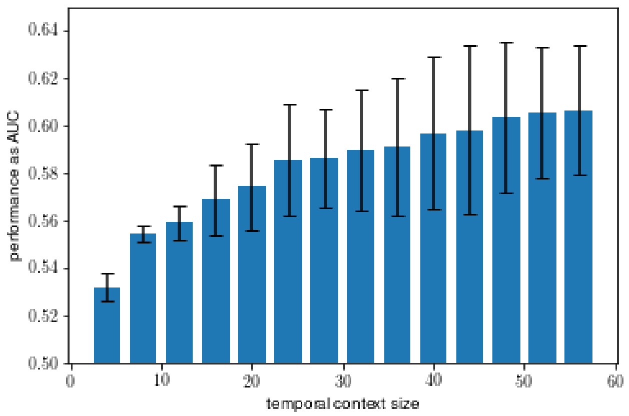

Figure 3. Results of temporal context size experiment.

2 C.2 which are for the track medians, while here all measurements are treated as independent). They show the relationship between the cells' velocity magnitude, shape, and size. The latter two cell features are measured by computing the cells' eccentricity and radiues of its Gaussian-like profile, respectively. Moreover, on each joint distribution, the maximum likelihood estimate (MLE) is shown with bold crosses and the median of the data with bold dots (where the numerical values can be found in Table 1b, 1c and 1d for Figure 2A,B and C respectively). In particular, the following insights are elaborated based on the median values (since they represent a robust metric of central tendency of the data):

observable	CD20	CD3 single	PD1	CD20	CD3 median per track	PD1
count	125161	136192	121230	2476	2805	2648
mean	2.007	2.063	2.548	1.646	1.724	2.117
std	2.049	2.111	2.245	0.972	1.059	0.970
min	0.002	0.001	0.002	0.087	0.101	0.112
25%	0.619	0.620	0.974	0.865	0.868	1.405
50%	1.327	1.349	1.888	1.508	1.515	2.038
75%	2.669	2.781	3.408	2.241	2.374	2.672
max	24.490	23.605	24.059	7.768	6.630	6.890

(a) Velocity magnitude statistics raw vs. per track (in μm/min)

class	corr	pval	mle_x	mle_y	median_x	median_y
CD20	0.041	1.23e-47	0.179	0.578	0.253	1.327
CD3	0.018	4.15e-11	0.131	0.578	0.214	1.349
PD1	0.015	2.1e-07	0.131	0.978	0.201	1.888

(b) eccentricity vs velocity [μm/min]

class	corr	pval	mle_x	mle_y	median_x	median_y
CD20	0.148	0	2.819	0.578	2.756	1.327
CD3	0.119	0	2.928	0.578	2.770	1.349
PD1	0.147	0	2.971	0.978	2.897	1.888

(c) diameter [μm] vs velocity [μm/min]

class	corr	pval	mle_x	mle_y	median_x	median_y
CD20	0.168	0	0.247	2.884	0.253	2.756
CD3	0.069	1.66e-142	0.131	2.971	0.214	2.770
PD1	0.038	8.18e-41	0.152	3.015	0.201	2.897

(d) eccentricity vs diameter [μm]

Table 1. Table 1a velocity statistics for figure 1. Table 1b, 1c and 1d statistics for figure 2.

Figure 2A (*Velocity magnitude vs. eccentricity*) illustrates the relationship between the cells' velocity and shape. Thus, based on the median of the data, PD1 cells move faster than those corresponding to CD3 and CD20 markers. Regarding the cells' shape, the eccentricity is lower for cells stained with PD1 and CD3 than CD20. In other words, PD1 and CD3 cells are geometrically closer to a circle, while CD20 cells to an ellipse.

Figure 2B (*Velocity magnitude vs. diameter*) shows the relationship between the cells' velocity and size and suggest (based on the median of the data) that PD1 cells have a larger diameter than CD3 and CD20 cells. Moreover, a closer look reveals that CD3 cells have a slightly larger diameter than those corresponding to CD20. By extending these findings to the velocity dimension, it is possible to conclude that cells with a larger diameter (i.e., PD1) move faster than the other (i.e., CD3 and CD20). Figure 2C (*Diameter vs. eccentricity*) shows the relationship between the cells' size and shape, using the cells' diameter and eccentricity as proxy measures, respectively. In this illustration, we observe (using the median of the data) that the highest eccentricity and the smallest diameter corresponds to CD20 cells. Conversely, PD1 cells have the largest diameter and lowest eccentricity. In this feature space, the CD3 cells lie between the latter two cell types. Thus, we can conjecture that the cells' geometrical shape is best described by ellipses when they have a small diameter. Then, as this diameter increases, the cells' shape becomes more regular (i.e., a circle).

2 Extended supervised analysis

2.1 Examples of representation and model evaluation schema

In this section we will provide more intuition about the evaluation schema which involves predictions at pixel level. Here, we show a illustrative experimental results of 3D CNN (as described in the paper) in across patient setting (single fit) for following folds:

1. CD20 vs. PD1: one test patient (ID=463) with 3 videos (`CD20_CD35_PD1`), AUC = 0.959, shown in Figure 4a.
2. CD3 vs. PD1: one test patient (ID=459) with 3 videos (`CD3_CD35_PD1`), AUC = 0.655, shown in Figure 4b
3. CD20 vs. CD3: one test patient (ID=600) with 5 video (`CD20_CD35_CD3`), AUC = 0.416, shown in Figure 4c

As already pointed out in the paper, CD20 vs. PD1 is easiest and most stable, followed by CD3 vs. PD1 with slight over-fitting. A model classifying CD20 vs. CD3 overfits right from the beginning, which is inline with our findings in the main paper. All models were trained for 100 epochs with batch-size of 64 and temporal patch-size of 8x64x64. Numbers of training and validation steps per epoch where chosen such that the sampled spatio-temporal volume is equivalent the training and validation data respectively. All models were trained by minimizing binary crossentropy loss with Adam learning rate of .0001, to prevent over-fitting we used L2 kernel regularizer (weight .001) on all weights and Dropout (rate .25) after last convolutional layer and before global pooling. All models were applied to each video from the test set by a moving sliding window (with given patch size) and spatial and temporal overlap of 75% and 50% respectively (avoids border artifacts).

2.2 Investigation of temporal context size

As already mentioned in the main paper, it might seem contradictory that neither level 2 nor level 4 can identify pattern that generalize across patients even though both of them also have access to movement information. We investigated this apparent paradox by adjusting the maximum track length, see Figure 3. For this we trained model based on level 1 representation (i.e. long-term track features) with varying the temporal context size from 4 to 56 time steps by random sub-sampling of tracks such that the amount of information is roughly equal across experiments, i.e. the total number of time steps is constant. In order to allow for error quantification we repeated each experiment 100 times and report the mean (as bar plot) with associated standard deviation (as error bars). This experiment reveals, on the one hand, that the performance quickly degrades with reduced temporal context size (track length). This explains the weak performance of the level 2 classifier as a shallow CNN with a correspondingly very limited receptive field, which exploits mostly local movement characteristics. On the other hand, the classification performance begins to saturate at 60 time frames and hence is not to expected to increase drastically for even larger temporal context, which provides a post-hoc justification for limiting the recording time to 20 minutes.

2.3 Deeper insights

Here, we extend the analysis introduced in Figure 3 of the main paper, by mainly two aspects: (1) difference between inter- and intra-patient experiments and the resulting patterns (see Figure 5) and (2) clustering of hidden representations uncovering different error rates associated with specific patterns (see Figure 6).

For comparison, Figure 5a shows the same patches as in the main paper (i.e. from the associated inter-patient experiment), while Figure 5b shows the top true positives patches from the associated intra-patient experiment. Please note, overall, the appearance of most likely patches remain also in intra-patient settings, where PD1 (follicular T-helper-cells) have a undefined morphology with blurred intermediate intensities inside a dense micro-environment. CD3 (T-cells) show a small round morphology with clearly defined and densely stained surfaces located inside a sparser micro-environments and CD20 (B-cells) show highly connected surfaces with clearly defined cell bodies surrounded by other B-cells. In summary, patterns found in inter-patient experiments are comparable of those found in intra-patients experiments.

In order to gain a better understanding of the model's capabilities, we used unsupervised clustering in the domain of hidden representations (activations from the penultimate layer) to indentify sub-populations associated with different error rates and patterns. Figure 6 shows the results of this analysis, where Figure 6a and 6b is for inter and intra patients respectively. Each plot consists of three sub-plots, one for each cell type. Each sub-plot then consists of three rows, one for each cluster within given cell-type. Each row is labelled with frequency of each cluster and the associated true positve rate (i.e. the number of true positives within each cluster). Especially Figure 6a shows interesting shifts in error rates associated with cluster (e.g. for CD3 bottom cluster: the cell's morphology appears to be more blurred which is associated with PD1 leading to considerable drop in performance with only 28% true positive rate).

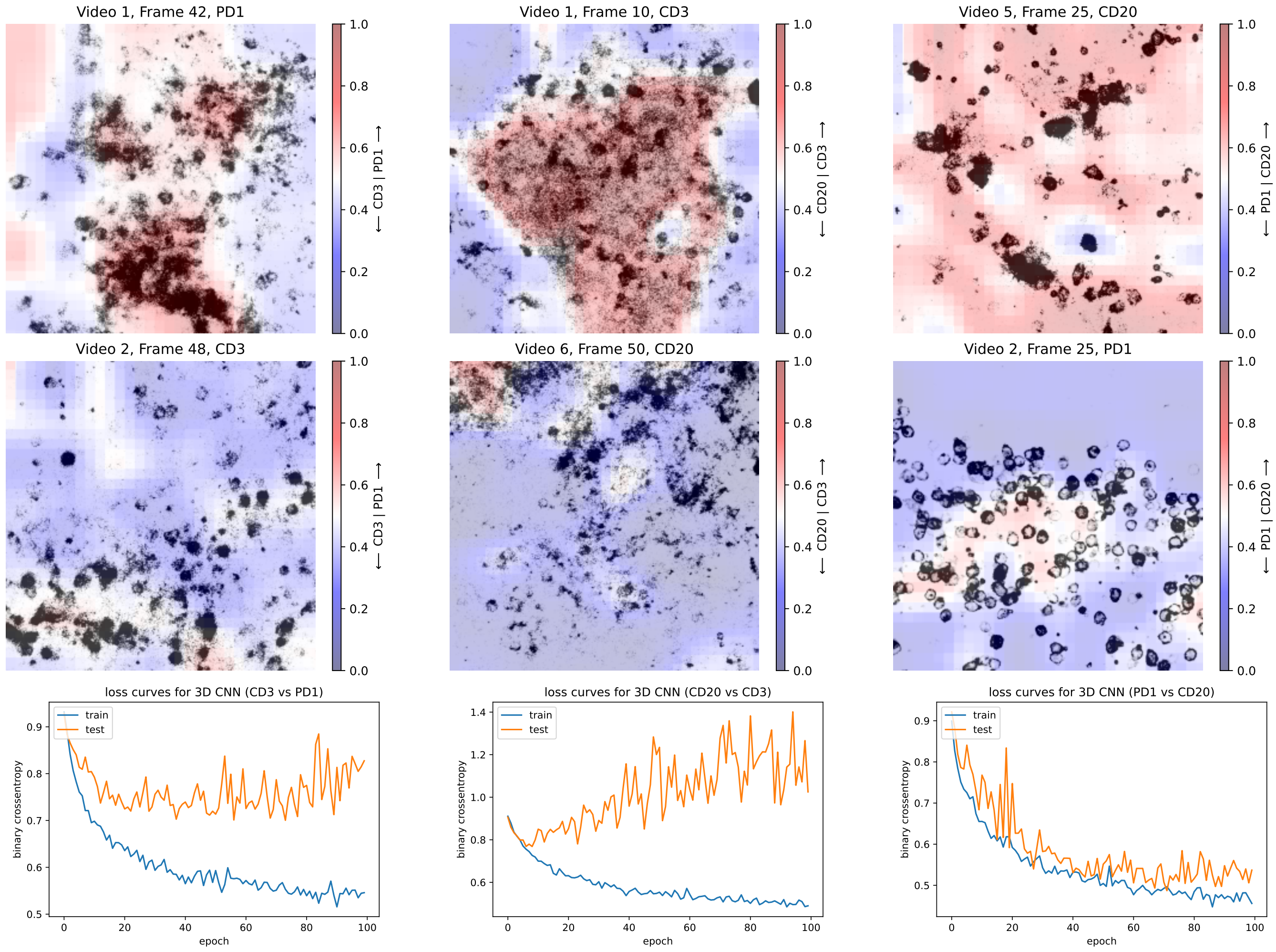

(a) CD3↔PD1 **(b)** CD20↔CD3 **(c)** CD20↔PD1

Figure 4. Illustrative experimental results of the experiments described in Section 2.1. Each column shows the learning curves (in terms of binary crossentropy loss) for both training and testing (please note over-fitting in some cases) at the bottom row. In the columns above we randomly picked a video and a frame from the test set for cell types considered in the specific experiment. The frame of the specific channel as gray-scaled background, on top the classifier output (in terms of probability belonging to either class) is visualized as heatmap above, where red is for the first and blue is for the second class (cell type). To get a idea of the temporal course, for each presented case we also provide the video files (as gif) in the supplementary information.

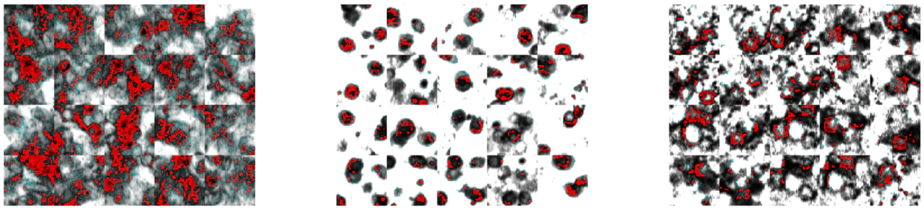

(a) PD1 (b) CD3 (c) CD20

(a) top true positives for inter patient experiments

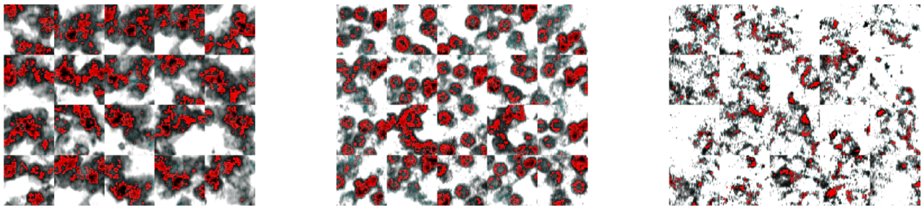

(a) PD1 (b) CD3 (c) CD20

(b) top true positives for intra patient experiments

Figure 5. Analysis of top true positive predictions showing 20 patches for each cell type, where Figure 5a and 5b is from inter and intra patients experiments respectively.

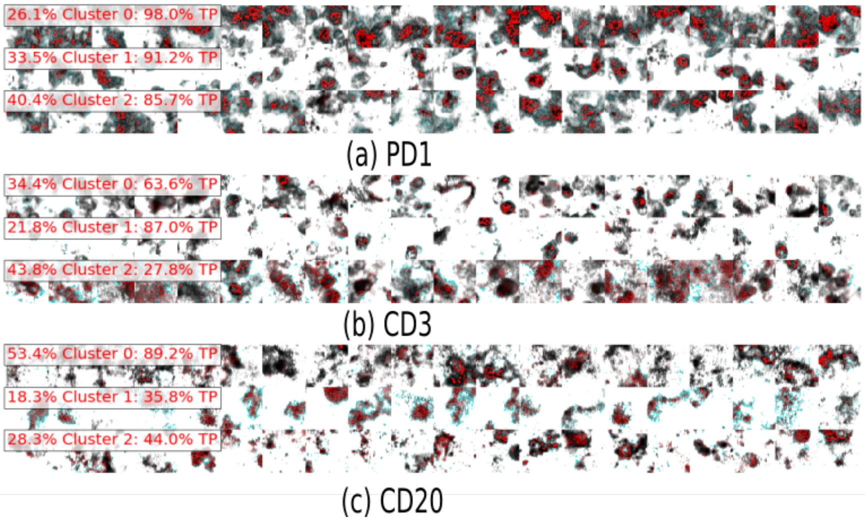

(a) inter patient

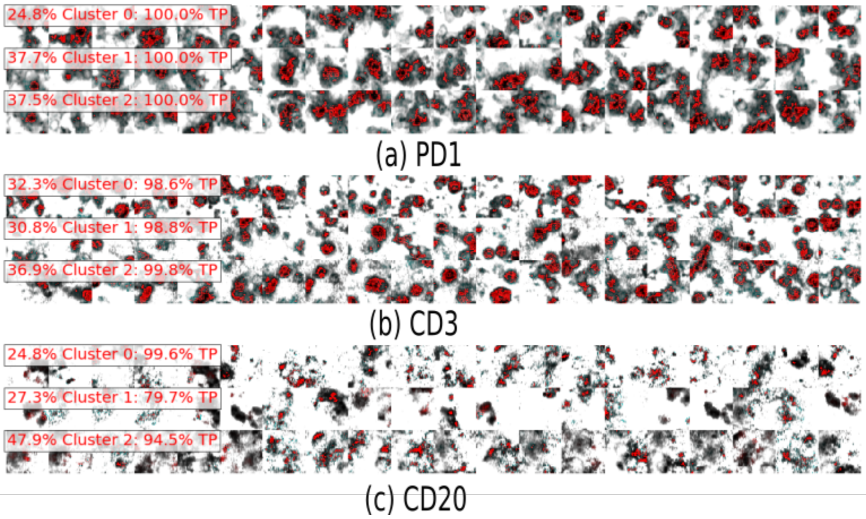

(b) intra patient

Figure 6. Results from error analysis by clustering hidden representations into three clusters using K-means.
