# Supplementary figures and images for: New definitions of human lymphoid and follicular cell entities in lymphatic tissue by machine learning
Source: Sci Rep. 2022 Nov 8;12:18991. doi: 10.1038/s41598-022-18097-9 (PMC9643435; doi:10.1038/s41598-022-18097-9)

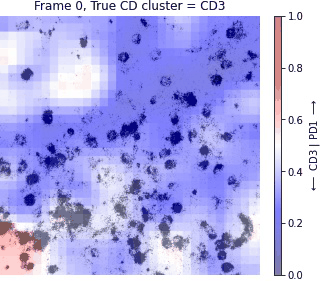

Supplement: Supplementary file 3 — Supplementary Figure 4. [file 41598_2022_18097_MOESM3_ESM.gif]

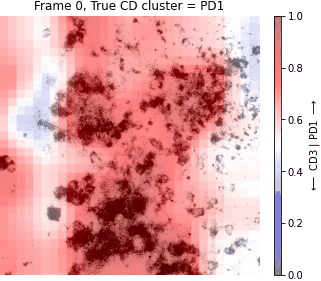

Supplement: Supplementary file 4 — Supplementary Figure 4. [file 41598_2022_18097_MOESM4_ESM.gif]

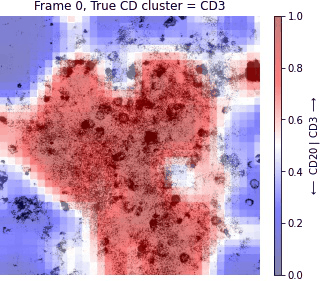

Supplement: Supplementary file 5 — Supplementary Figure 4. [file 41598_2022_18097_MOESM5_ESM.gif]

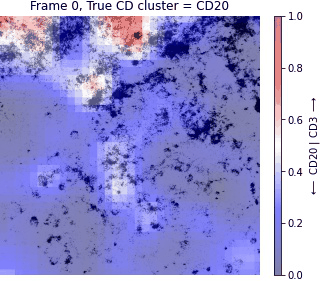

Supplement: Supplementary file 6 — Supplementary Figure 4. [file 41598_2022_18097_MOESM6_ESM.gif]

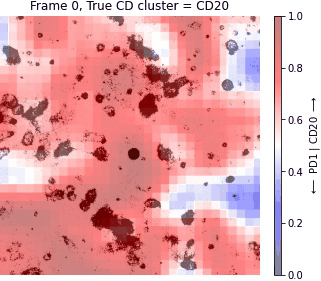

Supplement: Supplementary file 7 — Supplementary Figure 4. [file 41598_2022_18097_MOESM7_ESM.gif]

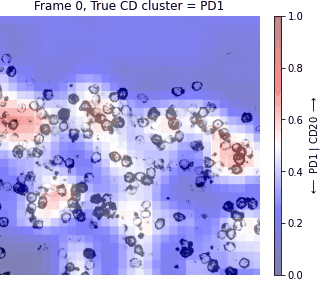

Supplement: Supplementary file 8 — Supplementary Figure 4. [file 41598_2022_18097_MOESM8_ESM.gif]
